# Correction to: The NAC Transcription Factor SlNAP2 Regulates Leaf Senescence and Fruit Yield in Tomato

**DOI:** 10.1093/plphys/kiae178

**Published:** 2024-05-24

In December 2023, the authors were alerted via PubPeer (https://pubpeer.com/publications/4C0F248FBC6644899610F9B28A6940) to errors in Figure 5C. Two leaf images in the original figure, framed in blue and magenta, were incorrectly placed and have been corrected in the revised figure below. The figure caption has also been updated accordingly. Additionally, for the sake of clarity, the authors have added the full set of 0 d control of Mock and ABA treatment.

**Figure 5C.**
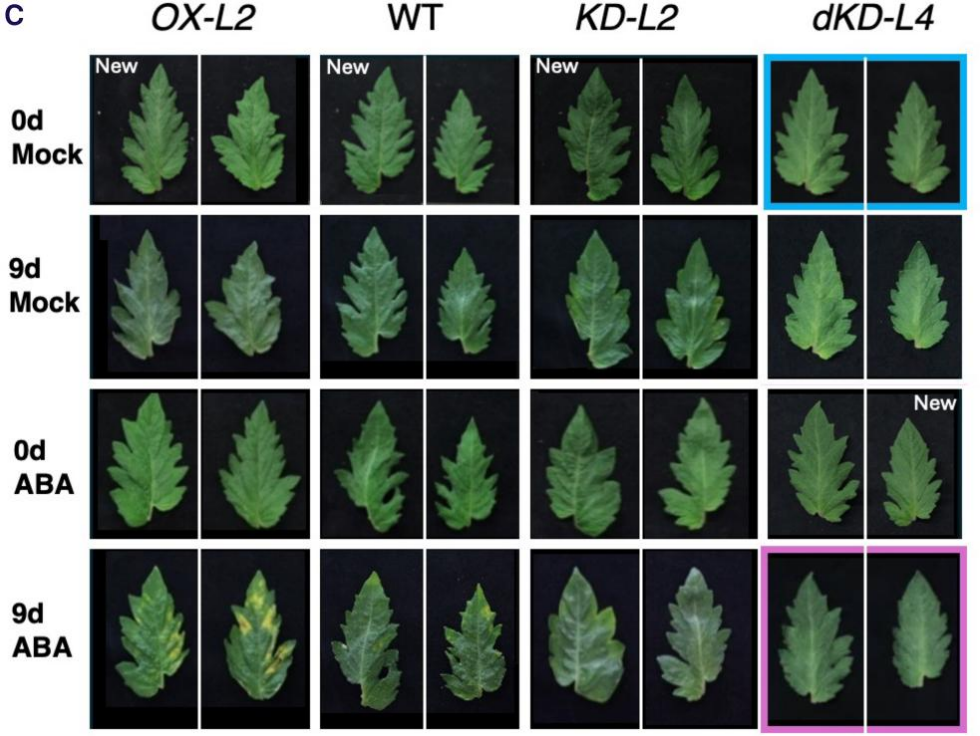
The phenotype of detached leaves from 10-week-old wild-type and *SlNAP2*-transgenic plants following treatment with 0.1% dimethyl sulfoxide (DMSO) solution (Mock) or 40 µM ABA, alongside their corresponding 0 d controls (the first row represents the state prior to the Mock treatment, and the third row represents the state before the ABA treatment).

The corresponding author was unable to reach the fourth co-author about this correction, but all other authors agree with it and apologize for these errors, which affect neither the conclusions drawn nor the quantitative data presented in the other panels within Figure 5.

